# Proteomic and immunocytochemical analyses of squamous components in two- and three-dimensional-cultured pancreatic ductal adenocarcinoma cell lines

**DOI:** 10.3892/ol.2026.15540

**Published:** 2026-03-19

**Authors:** Yuuki Shichi, Hiroki Tsumoto, Masakazu Fujiwara, Keisuke Nonaka, Yasuko Hasegawa, Seiichi Shinji, Hirofumi Rokutan, Kimimasa Takahashi, Tomio Arai, Yuri Miura, Toshiyuki Ishiwata

**Affiliations:** 1 Division of Aging and Carcinogenesis, Tokyo Metropolitan Institute for Geriatrics and Gerontology, Itabashi, Tokyo 173-0015, Japan; 2 Proteome Research, Tokyo Metropolitan Institute for Geriatrics and Gerontology, Itabashi, Tokyo 173-0015, Japan; 3 Department of Gastroenterological Surgery, Nippon Medical School, Bunkyo, Tokyo, 113-8603, Japan; 4 Department of Pathology, Tokyo Metropolitan Institute for Geriatrics and Gerontology, Itabashi, Tokyo 173-0015, Japan; 5 Department of Veterinary Pathology, School of Veterinary Medicine, Nippon Veterinary and Life Science University, Musashino, Tokyo 180-8602, Japan

**Keywords:** pancreatic cancer, proteomic analysis, immunocyto-chemical analysis, three-dimensional culture, p40

## Abstract

Adenosquamous carcinoma of the pancreas consists of both adenocarcinoma and squamous cell carcinoma components and shows a worse prognosis compared with pancreatic ductal adenocarcinoma (PDAC). Given that squamous cells are absent in healthy pancreatic tissue, their origin in pancreatic tumors remains unclear. The present study aimed to analyze ductal and squamous marker expression in PDAC cell lines under two-dimensional (2D) and three-dimensional (3D) culture conditions. A total of eight PDAC cell lines were examined using liquid chromatography-tandem mass spectrometry-based proteomics and immunocytochemistry to evaluate the expression of cytokeratins (CK7, CK20 and CK5/6), p63 and p40. CK7 was found to be expressed under both culture conditions in epithelial-type PDAC cell lines but not in mesenchymal-type PDAC cell lines. CK20 was not detected in any PDAC cell line under either culture condition. Among the squamous cell markers, CK5/6 was expressed in PK-59, PK-1 and T3M-4 cells in 2D culture, but not in PK-59 cells in 3D culture. Furthermore, p63 and its isoform p40 were strongly expressed in PK-8, PK-1 and T3M-4 cells in 2D culture but were not expressed in PK-8 cells in 3D culture. These squamous cell markers were not observed in mesenchymal PDAC cell lines, except for p63 in MIA PaCa-2 cells. Epithelial PDAC cells maintained their CK7^+^/CK20^−^ adenocarcinoma characteristics, while a number of cell lines also acquired squamous features. Overall, the present study provides new insight into the squamous cell component of PDAC and may therefore contribute to understanding the mechanism of its formation.

## Introduction

Pancreatic ductal adenocarcinoma (PDAC) is one of the most malignant cancers, with a 5-year overall survival rate of ~10%, and is characterized by extensive cellular heterogeneity and resistance to treatment (1–6). A number of PDAC cell lines specifically express epithelial proteins, whereas other cells lines express mesenchymal proteins (7–9) and the difference in sphere morphology, proliferative capacity, migratory capacity and anticancer drug resistance of epithelial and mesenchymal PDAC cell lines, becomes more apparent when cultured in three-dimension (3D) (10–14).

PDACs rarely contain squamous cell carcinoma components that proliferate in sheets and stain positive for cytokeratin (CK) 5, 6, p63 or p40 upon immunohistochemical staining. Adenosquamous carcinoma (ASC) is diagnosed when squamous cell components are found in >30% of pathological tissue specimen (15). ASC is observed in <1% of patients with PDAC, but it shows resistance to anticancer drugs and has a worse prognosis compared with that of PDAC (16–21). In addition to its aggressive clinical behavior, pancreatic ASC shares major driver mutations with conventional PDAC, including frequent tumor protein (TP)-53, KRAS and CDKN2A alterations (22). However, these common genetic traits are accompanied by alterations that affect chromatin regulators and epigenomic control, which distinguish ASC from typical PDAC. These observations suggest that ASCs share the same tumor lineage as PDAC but subsequently acquire additional molecular programs that promote squamous differentiation and therapeutic resistance.

Squamous cells are not present in the healthy pancreas, thus the origin of the squamous components in PDAC is unclear. To date, numerous hypotheses have been proposed regarding the origin of the squamous epithelium, including differentiation, squamous metaplasia and collision theories (23–26). In 3D culture systems, the intercellular interactions and concentration gradients of oxygen and nutrients enable the reproduction of an environment closer to the tumor microenvironment *in vivo* compared with that achieved through a traditional two-dimensional (2D) culture (27,28). However, to the best of our knowledge, the effects of 3D culture on the expression and spatial distribution of ductal and squamous epithelial markers in PDAC cells have not been systematically investigated.

In the present study, the aim was to investigate the expression profiles of ductal or squamous components in a panel of epithelial and mesenchymal PDAC cell lines grown in 2D and 3D cultures using proteomic and immunocytochemical analyses.

## Materials and methods

### Cell culture

Human PDAC cell lines PK-8 (cat. no. RCB2700), PK-45P (cat. no. RCB2141), T3M-4 (cat. no. RCB1021) and KP4 (cat. no. RCB1005) were provided by RIKEN BioResource Research Center through The National Bio-Resource Project of the Ministry of Education, Culture, Sports, Science and Technology and The Japan Agency for Medical Research and Development (29). Further human PDAC cell lines PK-59 (cat. no. TKG0492), PK-1 (cat. no. TKG0239), PANC-1 (cat. no. TKG0606) and MIA PaCa-2 (cat. no. TKG0227) were obtained from The Cell Resource Center for Biomedical Research through the Institute of Development, Aging and Cancer of Tohoku University (Sendai, Japan). Previous studies describing the establishment of PK-1 and T3M-4, as well as the other PDAC cell lines used in the present study, have indicated that they were derived from patients with histologically confirmed pancreatic adenocarcinoma (30,31). Cells were cultured in RPMI-1640 medium (Nacalai Tesque, Inc.) supplemented with 10% FBS (Gibco; Thermo Fisher Scientific, Inc.) at 37°C in a humidified 5% CO_2_ atmosphere. Using a *Mycoplasma* PCR Detection Kit (cat. no. 25239, iNtRON Biotechnology) according to the manufacturer's instructions, it was determined that none of the cells exhibited *Mycoplasma* contamination. Genomic DNA was extracted from PDAC cells using the DNeasy Blood and Tissue Kit (Qiagen), following the manufacturer's protocol. Short tandem repeats were analyzed using the GenePrint^®^ 10 System (Promega Corporation), following the manufacturer's protocol. All PDAC cell lines were correctly genotyped and showed no evidence of contamination.

### Proteomic analysis

For the 2D culture, adherent cells were collected by trypsinization, centrifuged (170 × g; 5 min; 20°C) and washed with PBS. This procedure was performed twice. For 3D cultures, PDAC cells were seeded at 3.0×10^3^ cells/well in low-attachment 96-well plates (Thermo Fisher Scientific, Inc.) for 7 days. The detailed protocols for 3D sphere formation were conducted as previously described (12).

### Protein extraction

Cell pellets from 2D and 3D cultures were suspended in 100 µl lysis buffer [5% SDS; 50 mM triethylammonium bicarbonate (TEAB; Thermo Fisher Scientific, Inc.) buffer; pH 8.5], sonicated and centrifuged (16,000 × g for 10 min at room temperature). The supernatants were collected, and protein concentrations were measured using Pierce 660 nm Protein Assay Reagent (Thermo Fisher Scientific, Inc.) with Ionic Detergent Compatibility Reagent (Thermo Fisher Scientific, Inc.).

### Trypsin digestion

Supernatants containing 30 µg protein were diluted with lysis buffer up to 23 µl and digested by trypsin (Promega Corporation) using a S-Trap™ Micro Spin column (ProtiFi, LLC), according to the manufacturer's instructions with minor modifications. Briefly, samples were reduced with 5 mM tris(2-carboxyethyl)phosphine (Thermo Fisher Scientific, Inc.) at 55°C for 15 min, alkylated with 20 mM iodoacetamide (Thermo Fisher Scientific, Inc.) at 25°C for 20 min in the dark and digested with 3 µg trypsin (protein/trypsin, 10:1) at 47°C for 2 h. Eluates were evaporated *in vacuo* to dryness and dissolved in 100 mM TEAB buffer (pH 8.5). The sample corresponding to 5 µg protein was desalted using GL-Tip SDB (GL Sciences Inc.) and dissolved in 5% acetonitrile (MeCN; FUJIFILM Wako Pure Chemical Corporation) containing 0.1% formic acid (FA; FUJIFILM Wako Pure Chemical Corporation) at a concentration of 0.25 µg/µl.

### Liquid chromatography-tandem mass spectrometry analysis

Liquid chromatography-tandem mass spectrometry analysis of tryptic peptides was performed using the Vanquish Neo coupled with the Orbitrap Fusion Lumos mass spectrometer (Thermo Fisher Scientific, Inc.) by injecting 2 µl sample (0.5 µg protein). The PepMap™ Neo Trap Cartridge (0.3×5.0 mm; 5 µm particle size; C18; Thermo Fisher Scientific, Inc.) and NANO HPLC Capillary column (75 µm × 12 cm; 3 µm particle size; C18; Nikkyo Technology, Co., Ltd.) were used as the trap and analytical columns, respectively. Peptide separation was performed using water containing 0.1% FA (solvent A) and MeCN containing 0.1% FA (solvent B) at a flow rate of 300 nl/min. The linear gradient for peptide separation was as follows: 0–1 min, 5% B; 1–91 min, 5–22% B; 91–96 min, 22–40% B; 96–100 min, 40–90% B; and 100–110 min, 90% B. The mass spectrometer was operated in data-dependent acquisition mode. All mass spectra (MS1) were acquired with the following settings: Spray voltage, 2.0 kV; ion transfer tube temperature, 275°C; detector type, orbitrap; resolution, 60,000; mass range, normal; scan range, 350–1,500 m/z; radiofrequency lens, 30%; automatic gain control (AGC) target, standard; maximum ion injection time (IT), auto; and polarity, positive. All MS/MS spectra (MS2) were acquired using the following settings: Isolation mode, quadrupole; isolation window, 1.6 m/z; activation type, higher-energy collisional dissociation (HCD); collision energy mode, fixed; HCD collision energy type, normalized; HCD collision energy, 30%; detector type, orbitrap; orbitrap resolution, 15,000; mass range, normal; scan range mode, auto; AGC target, standard; maximum IT mode, auto; dynamic exclusion, 20 sec; and charge states, 2–7.

### Data analysis

Protein identification and label-free quantification (LFQ) were performed using the Proteome Discoverer software (version 3.0; Thermo Fisher Scientific, Inc.). The parameters for protein identification were as follows: Search engine, CHIMERYS (version 1.7; MSAID GmbH); fragment mass tolerance, 0.02 Da; enzyme, trypsin (full); maximum missed cleavage sites, 2; static modification, carbamidomethyl (Cys, +57.021 Da); dynamic modification, oxidation (Met, +15.995 Da); and protein database, *Homo sapiens* (20,426 proteins downloaded on 09-20-2023 from UniProtKB; http://www.uniprot.org/uniprotkb). The parameters for LFQ were as follows: Precursor quantification, area; normalization mode, total peptide amount. The results of the proteomic analysis of ductal and squamous cell markers are summarized in Table SI.

### Immunocytochemical analysis

Immunocytochemical analysis was performed as previously described (12,32). Adherent PDAC cells were collected after trypsin treatment, centrifuged (170 × g, 5 min, 20°C) and fixed with 10% neutral-buffered formalin for 3 h at 22°C. For sphere formation in 3D culture, PDAC cells were seeded in a growth medium consisting of RPMI-1640 medium (Nacalai Tesque) supplemented with 10% FBS at 3.0×10^3^ cells/well in 96-well low-attachment plates (cat. no. 174925; Thermo Fisher Scientific, Inc.). After 7 days, the spheres were aspirated using micropipettes and fixed in formalin for 3 h at 22°C. Formalin was removed using a micropipette and both PDAC cells and spheres were dehydrated in graded ethanol and embedded in paraffin. Subsequently, serial sections of the cell blocks (3 µm thick) were stained using the BOND Polymer Refine Detection kit (Leica Biosystems) according to the manufacturer's instructions.

Mouse monoclonal anti-CK7 (cat. no. 713481; ready-to-use) and anti-p40 (cat. no. 418171; ready-to-use) antibodies were purchased from Nichirei Biosciences, Inc. Mouse monoclonal anti-CK5/6 (cat. no. M7237; 1:1,000), anti-p63 (cat. no. M7317; 1:50) and anti-CK20 (cat. no. M7019; 1:5,000) antibodies were purchased from Dako; Agilent Technologies, Inc. Furthermore, rabbit monoclonal anti-ATP binding cassette subfamily C (ABCC)-1 (cat. no. D5C1X; 1:100), anti-MMP-2 (cat. no. D4M2N; 1:100), anti-MMP-14 (cat. no. E3S5S; 1:100) and anti-ATP-binding cassette sub-family G (ABCG)-2 (cat. no. D5V2K; 1:200) antibodies were purchased from Cell Signaling Technology, Inc. and the rabbit monoclonal anti-ABCC2 (anti-MRP2; cat. no. EPR10998; 1:1,000) antibody was purchased from Abcam. For CK7 and CK20, antigen retrieval was performed using the Bond Enzyme Pretreatment Kit (cat. no. AR9551; Leica Biosystems) according to the manufacturer's instructions. For CK5/6, p63, p40, ABCC1, ABCC2, ABCG2, MMP-, and MMP-14, antigen retrieval was performed using the BOND Epitope Retrieval Solution 2 (cat. no. AR964; Leica Biosystems) according to the manufacturer's instructions. Tissue sections were incubated with each primary antibody for 15 min at 22°C. Antigen detection was performed using DAB, followed by counterstaining with hematoxylin for 2 min at 22°C. Negative controls were generated by omitting the primary antibodies. Images were captured using the PhenoImager Mantra™ 2 multispectral microscope (Akoya Biosciences, Inc.).

### Pathway analysis

Pathway analysis was performed using KeyMolnet software (version 6.2; KM Data Inc.) (33,34). To identify the molecular pathways associated with squamous metaplasia-related phenotypes, PK-45P was selected as the reference epithelial PDAC cell line, as immunocytochemical analysis demonstrated the absence of p40 expression under 2D and 3D culture conditions. Using PK-45P as the denominator, pathway analyses were conducted for PK-1 and T3M-4 cells, which exhibited squamous metaplasia-associated features. Proteomic datasets obtained through label-free quantitative proteomic analysis were used as input for pathway analysis. The list of identified proteins with abundance ratios and P-values was imported into KeyMolnet software and pathway analysis was performed using the ‘interrelation search’ algorithm to generate molecular interaction networks within a single path from the input molecules, including direct activation/inactivation and transcriptional activation/repression. Pathways associated with the input protein sets were extracted from the curated KeyMolnet knowledge base.

## Results

### Proteomic and immunocytochemical analyses of CK7 in PDAC cells

**Figure 1.**
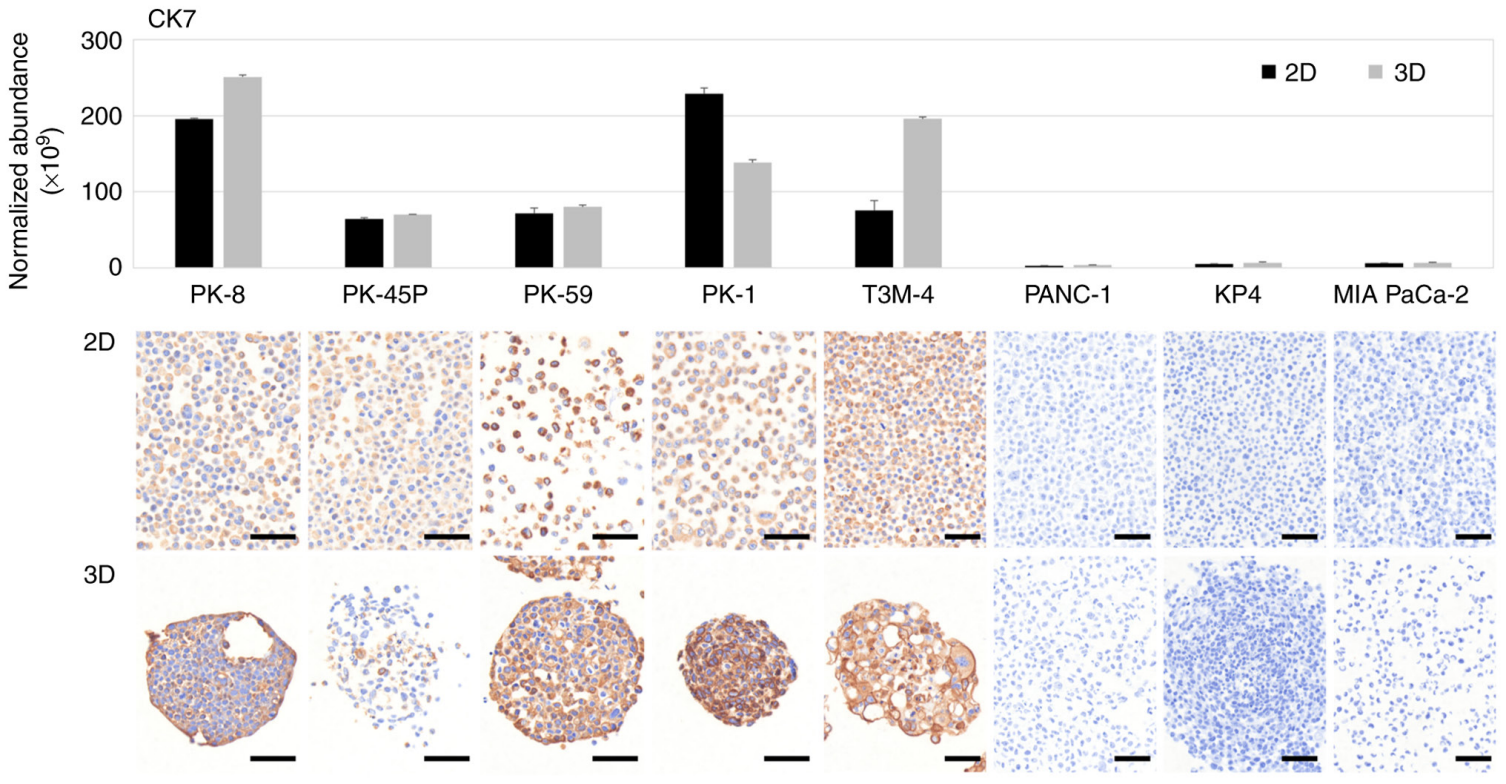
Proteomic and immunocytochemical analyses of cytokeratin CK7. The expression of CK7 in 2D and 3D-cultured five epithelial (PK-8, PK-45P, PK-59, PK-1 and T3M-4) and three mesenchymal (PANC-1, KP4 and MIA PaCa-2) pancreatic ductal adenocarcinoma cell lines analyzed through proteomics and immunocytochemistry. The upper panel indicates proteomic analysis, with the lower panels exhibiting immunocytochemical staining. Scale bars, 50 mm. CK, cytokeratin; 2D, two-dimensional; 3D, three-dimensional.

Proteomic and immunocytochemical analyses were performed using five PDAC cell lines exhibiting epithelial properties with high E-cadherin and low vimentin expression (PK-8, PK-45P, PK-59, PK-1 and T3M-4) and three PDAC cell lines (PANC-1, KP4 and MIA PaCa-2) exhibiting the opposite expression pattern (12). PK-8, PK-45P, PK-59, PK-1, T3M-4, PANC-1, KP4 and MIA PaCa-2 were listed in descending order of E-cadherin mRNA levels, as previously assessed using quantitative real-time PCR analysis in our previous study (12) (Fig. 1). Proteomic analysis of PDAC cells cultured in 2D and 3D showed that CK7, an intermediate filament found in ductal cells and highly expressed in pancreatic cancer, was detected in five epithelial PDAC cell lines but was expressed at lower levels in three mesenchymal PDAC cell lines (Fig. 1, upper panel). To further determine the results of the proteomic analysis, cell blocks of 2D and 3D cultured PDAC cells were prepared and stained with a specific antibody against CK7. Immunocytochemical staining showed that CK7 was positive in all epithelial PDAC cell lines cultured in 2D and 3D but negative in mesenchymal PDAC cell lines (Fig. 1, lower panels). By contrast, CK20 was not detected above the proteomic analysis threshold in any PDAC cell line cultured in 2D or 3D conditions and was consistently shown to be negative using immunocytochemistry (Fig. S1).

### Proteomic and immunocytochemical analyses of CK5/6 in PDAC cells

**Figure 2.**
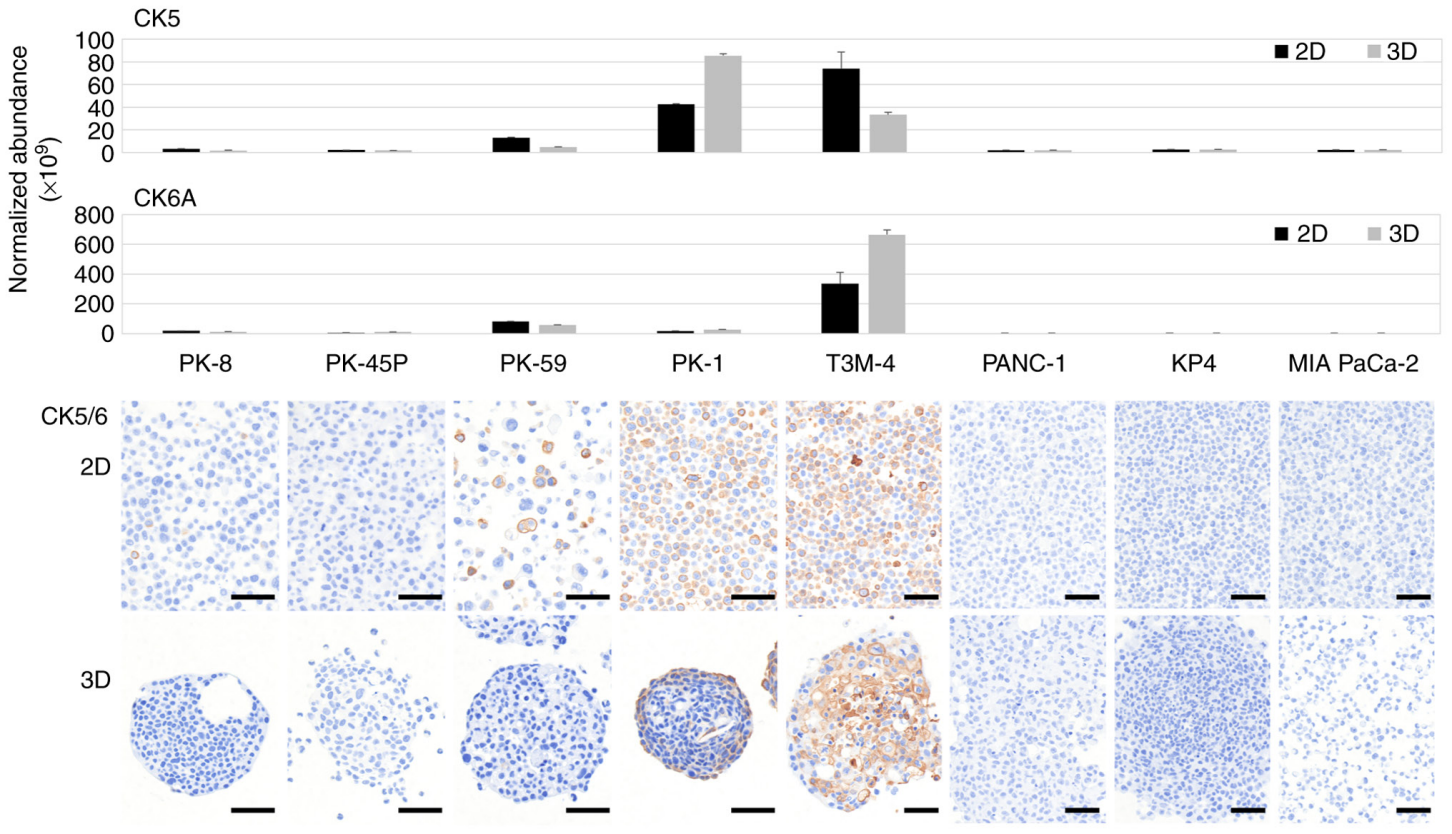
Proteomic and immunocytochemical analyses of CK5 and CK6A. The expression levels of CK5 and CK6A in eight different pancreatic ductal adenocarcinoma cell lines cultured in 2D and 3D media were examined using proteomic analysis. In addition, specific antibodies for CK5/6 were used to demonstrate their expression in pancreatic ductal adenocarcinoma cell lines cultured in 2D and 3D. The upper panels indicate proteomic analysis and the lower panels exhibit immunocytochemical staining. Scale bars, 50 mm. CK, cytokeratin; 2D, two-dimensional; 3D, three-dimensional.

CK5 is a product of the keratin (KRT)-5 gene, whereas CK6A, CK6B and CK6C are products of KRT6A, KRT6B and KRT6C, respectively. CK5/6 is not expressed in PDAC cells but is expressed in squamous carcinomas (35,36). Proteomic analysis revealed that CK5 was highly expressed in PK-1 and T3M-4 cells, whereas CK6A was highly expressed in T3M-4 cells only (Fig. 2, upper panels). Low levels of CK6B and CK6C were detected, with a slight increase observed in T3M-4 cells (Fig. S2). No increase in the expression of these CKs was observed in the mesenchymal PDAC cell lines. Immunocytochemical staining of the cell blocks showed diffuse CK5/6 localization in the majority of PDAC cells in 2D cultured PK-1 and T3M-4 cell lines. By contrast, in 3D cultures, CK5/6 was localized to the periphery of the sphere of PK-1 cells and the center of the sphere of T3M-4 cells (Fig. 2, lower panels).

### Proteomic and immunocytochemical analyses of p63 in PDAC cells

**Figure 3.**
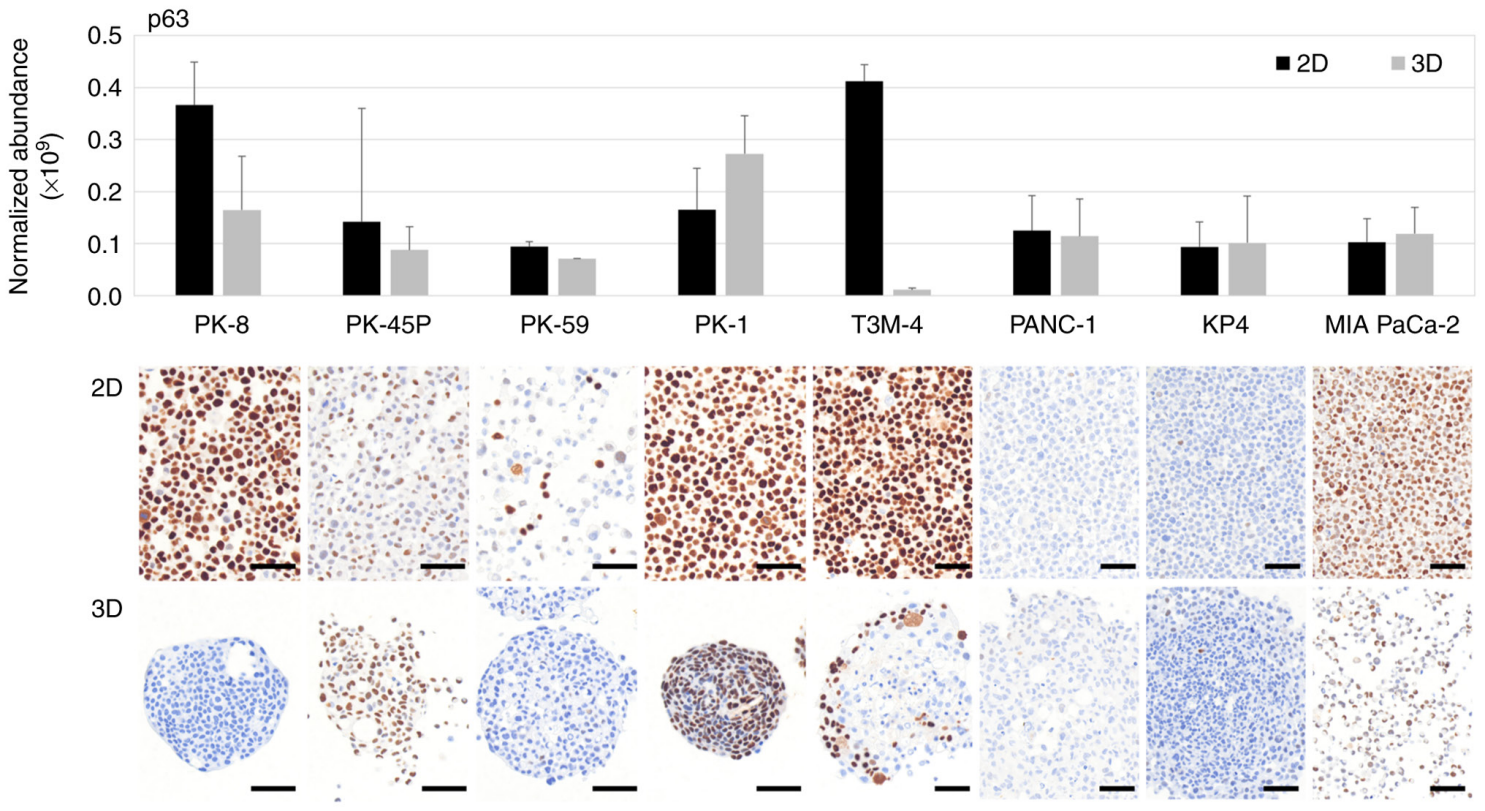
Proteomic and immunocytochemical analyses of p63 in pancreatic ductal adenocarcinoma cell lines. The expression of p63 in eight pancreatic ductal adenocarcinoma cell lines cultured in 2D and 3D cultures analyzed through proteomics and immunocytochemistry. The upper panel indicates proteomic analysis with the lower panels exhibiting immunocytochemical staining. Scale bars, 50 mm. 2D, two-dimensional; 3D, three-dimensional.

p63 is transcribed from the TP63 gene and p40 is one of its isoforms generated through alternative promoter usage (37). Proteomic analysis revealed that p63 was highly expressed in PK-8 and T3M-4 cells under 2D culture conditions, whereas relatively higher p63 expression was observed in PK-1 cells under 3D culture conditions (Fig. 3, upper panel). Notably, due to the extensive shared peptide sequences between p63 isoforms, liquid chromatography mass spectrometry-based proteomic analysis does not discriminate between the transactivating p63 (Tap63) and dominant-negative (ΔNp63; p40) isoforms. Immunocytochemical staining showed that p63 was positive in the majority of PK-8 cells cultured in 2D but was negative when cultured in 3D (Fig. 3, lower panels). Furthermore, p63 was present in the majority of PK-1 cells in 2D and 3D cultures. In T3M-4 cells cultured in 3D, only the cells at the periphery of the spheres were found to be p63-positive. In addition, a small number of p63-positive cells were found in PK-45P and MIA PaCa-2 cells in 2D and 3D cultures and in PK-59 cells in 2D culture. Although p63 immunoreactivity was occasionally observed in MIA PaCa-2 cells, these cells were consistently negative for p40 expression and demonstrated no elevation in p63 levels by proteomic analysis, indicating the absence of bona fide squamous differentiation.

### Immunocytochemical analysis of p40 in PDAC cells

**Figure 4.**
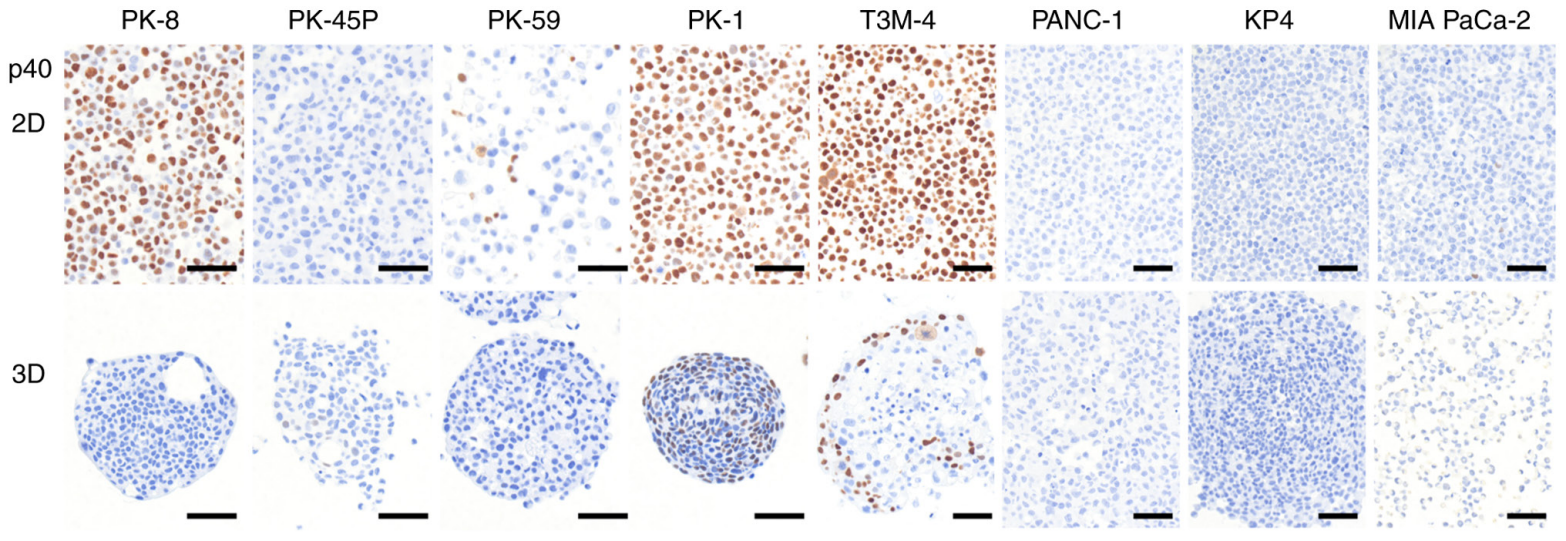
Immunocytochemical analysis of p40 in PDAC cell lines. The localization of p40 in PDAC cell lines cultured in 2D and 3D cultures was examined by immunocytochemical staining with a specific antibody. The upper panel indicates the 2D culture with the lower panel exhibiting the 3D culture. Scale bars, 50 mm. PDAC, pancreatic ductal adenocarcinoma; 2D, two-dimensional; 3D, three-dimensional.

p40 is considered a more specific marker than p63 in squamous cell carcinoma, however it cannot be detected by proteomic analysis due to structural overlaps (38). p40 was detected by immunocytochemical staining in PK-8, PK-1 and T3M-4 cells cultured in 2D (Fig. 4, upper panel). In 3D culture, PK-8 cells were p40-negative, whereas PK-1 and T3M-4 cells located at the periphery of the spheres were p40-positive (Fig. 4, lower panel). Proteomic analysis detected p63-related signals in PK-8 cells cultured under 3D conditions; however, p40 expression was not observed by immunocytochemical analysis under the same conditions. This discrepancy indicates that proteomic detection of p63 does not necessarily reflect p40 (ΔNp63) expression in 3D-cultured PK-8 cells. Immunocytochemical analyses of MMP-14 and ABCC1 revealed positive immunoreactivity predominantly in cells at the periphery of spheres exhibiting squamous metaplasia, as indicated by p40 positivity (Fig. S3). By contrast, ABCC2, ABCG2 and MMP-2 showed no detectable immunoreactivity in either the sphere core or peripheral regions (Fig. S3).

### Pathway analysis of squamous metaplasia-associated proteomic profiles

**Figure 5.**
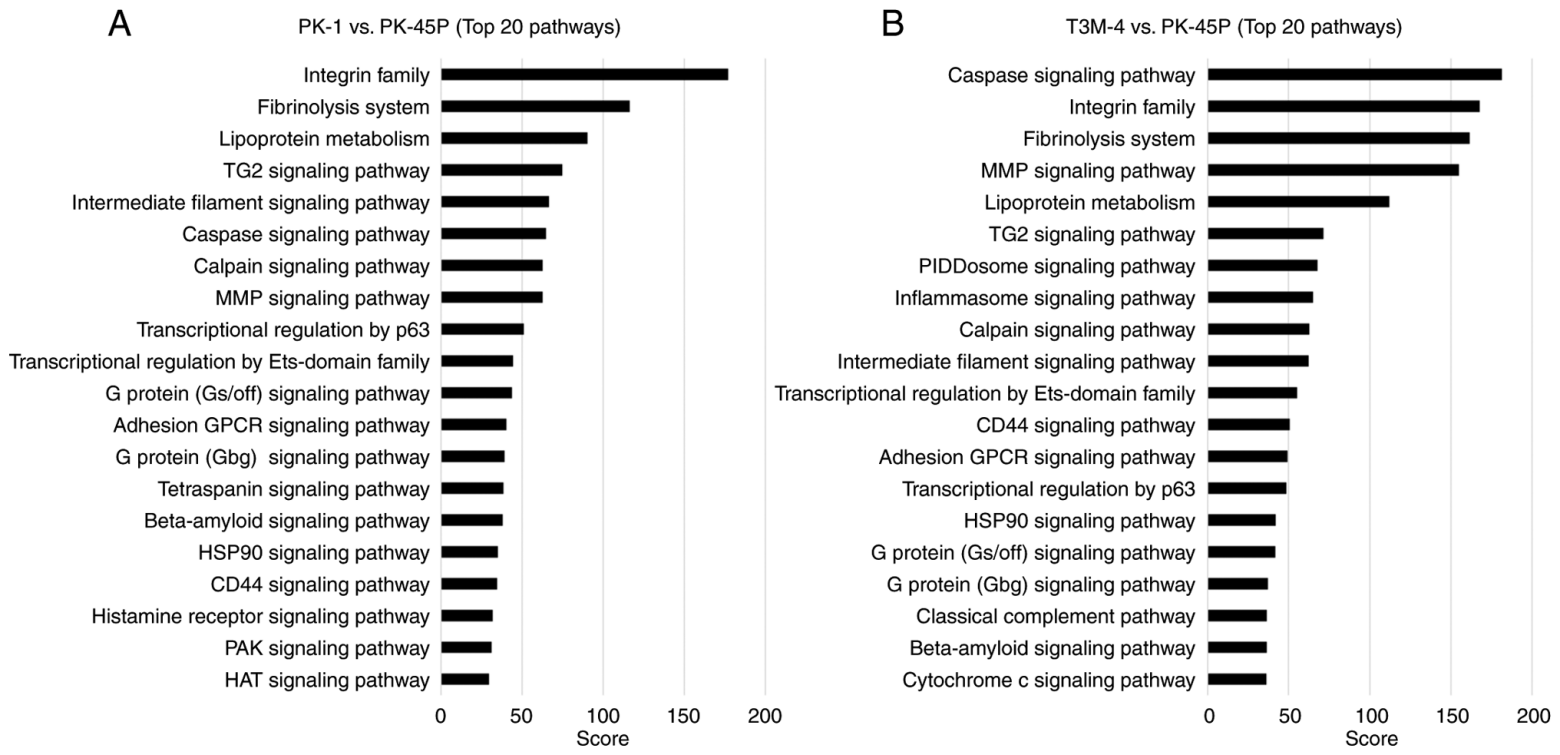
Pathway analysis comparing PK-1 and T3M-4 with PK-45P. (A) Top 20 enriched pathways in PK-1 relative to PK-45P. (B) Top 20 enriched pathways in T3M-4 relative to PK-45P. Horizontal bar graphs display pathways ranked by enrichment score with higher scores indicating stronger pathway enrichment.

To explore molecular pathways associated with squamous metaplasia-associated phenotypes in PDAC cell lines, pathway analysis was performed using KeyMolnet software based on proteomic comparisons of PK-1 or T3M-4 cells with the epithelial reference cell line PK-45P. The top 20 enriched pathways identified in each comparison are shown in Fig. 5A (PK-1/PK-45P) and Fig. 5B (T3M-4/PK-45P). In the PK-1/PK-45P comparison (Fig. 5A) pathways related to cell adhesion and extracellular matrix interactions, including the ‘integrin family’, ‘fibrinolysis system’ and the ‘MMP signaling pathway’, showed high enrichment scores. Pathways associated with the ‘intermediate filament signaling pathway’, the transglutaminase 2 ‘(TG2) signaling pathway’ and ‘transcriptional regulation by p63’ were also prominently enriched. In the T3M-4/PK-45P comparison (Fig. 5B), enrichment of pathways associated with cell adhesion and extracellular matrix remodeling, such as ‘integrin family’, fibrinolysis system’, ‘MMP signaling pathway’, ‘TG2 signaling pathway’ and ‘intermediate filament signaling pathway’, was observed. By contrast with PK-1 cells, T3M-4 cells exhibited marked enrichment of pathways associated with ‘caspase signaling pathway’, ‘PIDDosome signaling pathway’, ‘inflammasome signaling pathway’, ‘classical complement pathway’ and ‘cytochrome c signaling pathway’. Numerous pathways, including the ‘integrin family’, ‘fibrinolysis system’, ‘MMP signaling pathway’, ‘TG2 signaling pathway, ‘intermediate filament signaling pathway’ and ‘transcriptional regulation by p63’, were commonly enriched in PK-1 and T3M-4 cells. Collectively, these results indicate that PK-1 and T3M-4 cells share a core set of molecular pathways associated with squamous differentiation, while each cell line exhibits a distinct pathway enrichment profile.

## Discussion

Previously, it was reported that CK7, an indicator of pancreatic ductal differentiation, was expressed in PK-1 cells (epithelial PDAC cells) but not in PANC-1 cells (mesenchymal PDAC cells) in both 2D and 3D cultures (11). In the present study, four epithelial PDAC cell lines, in addition to PK-1 cells, expressed CK7, whereas two mesenchymal PDAC cell lines and PANC-1 cells did not express CK7. The majority of PDAC cells are characterized by being CK7-positive and CK20-negative and are expressed in the epithelial cells of the digestive tract, such as the large intestine (39). Epithelial PDAC cell lines exhibited characteristics of typical PDAC cells (CK7^+^/CK20^−^), whereas mesenchymal PDAC cells were CK7^−^/CK20^−^. This highlights the diversity of PDAC and emphasizes the need for caution in the pathological diagnosis of PDAC with mesenchymal features.

CK7 expression was restricted only to epithelial-type PDAC cell lines and was absent in mesenchymal-type cell lines. By contrast, squamous cell markers were detected only in a subset of epithelial-type PDAC cell lines, namely PK-1 and T3M-4, and were absent in mesenchymal-type PDAC cell lines. These findings indicated that squamous differentiation preferentially occurred in PDAC cells with a potential for pancreatic ductal epithelial differentiation, whereas mesenchymal-type PDAC cell lines lack both ductal and squamous differentiation features, representing a distinct differentiation state.

Next, the expression of CK5/6, p63 and p40 was examined, which are commonly used markers of squamous epithelial components in the pathological diagnosis of PDAC cell lines. CK5/6 expression has been reported in the basal cell types of pancreatic cancer, pancreatic cancer with squamous cell differentiation and ASC (40,41). p63 is a transcription factor belonging to the p53 family and two key (TAp63 and ΔNp63) are produced from the TP63 gene by an alternative promoter (37). p63 is expressed in squamous epithelial cells, squamous carcinoma cells, basal cells and cells with stem cell-like properties (42). ΔNp63, also known as p40 has been suggested to be more specific for squamous epithelial cells compared with p63 (43). In the present study, p63 and p40 were extensively upregulated in the majority of the epithelial PDAC cell lines, PK-1 and T3M-4, in 2D culture. By contrast, in 3D culture, p40 expression was observed only at the periphery of the spheres of these PDAC cells. In the PDAC cell lines PK-1 and T3M-4, three squamous cell markers were detected by both proteomics and immunohistochemistry, suggesting that these cell lines have the ability to produce squamous components.

Furthermore, no consistent changes in squamous marker expression were observed between 2D and 3D culture conditions. Some epithelial PDAC cell lines showed relatively uniform and high squamous marker expression throughout the cell population in 2D culture; however, under 3D culture conditions, squamous marker expression was either overall reduced or became spatially restricted to cells located at the periphery of the spheres, rather than being uniformly increased across all cells. Notably, 3D culture did not result in a global upregulation of squamous markers compared with 2D culture, but instead altered their spatial distribution in a cell line-dependent manner. These observations do not support the notion that 3D culture uniformly promotes squamous differentiation, but rather indicate that 3D culture modulates the localization of squamous marker expression without consistently enhancing squamous differentiation. Therefore, it cannot be concluded that 3D culture intrinsically induces squamous differentiation.

In addition, the technical and biological distinction between p63 and its ΔNp63 (p40) isoform should be considered. Although the p63 protein was detected using proteomic analysis, liquid chromatography-mass spectrometry does not discriminate between individual p63 isoforms due to extensive shared peptide sequences. By contrast, immunocytochemical analysis with isoform-specific antibodies revealed a dissociation between total p63 and ΔNp63 (p40) expression, as exemplified by PK-8 cells cultured under 3D conditions, which showed p63 detection by proteomics but lacked p40 by immunocytochemistry. This indicates that p63 positivity alone does not necessarily reflect activation of ΔNp63-driven squamous differentiation programs. On the surface of PK-1 cell spheres, scanning and transmission electron microscopy has revealed a flattened layer of cells (11,12). These cells may indicate the morphological differentiation of squamous epithelial cells. As the expression of squamous components differs between 2D and 3D cultures, these spatially distinct expression patterns suggest a role for intercellular interactions in the regulation of squamous differentiation.

Notably, the localization patterns of CK5/6 under 3D culture conditions differed between PK-1 and T3M-4 cells, although the precise mechanisms underlying this difference remain unclear. However, additional immunocytochemical analyses of proteins in relation to anticancer drug resistance and extracellular matrix degradation, revealed distinct spatial associations with CK5/6 localization. These findings suggest that the differential localization of CK5/6 may reflect distinct biological contexts or stages of squamous differentiation, potentially corresponding with structurally stabilized squamous metaplasia in PK-1 cells and a more stress-associated or dynamic differentiation process in T3M-4 cells. Furthermore, the spatial coincidence of CK5/6 expression with selected drug resistance- and extracellular matrix-related proteins indicates that squamous marker-expressing cells may possess enhanced adaptive properties, particularly at the periphery of tumor spheres. Consistent with these observations, the immunoreactivity of MMP-14 and ABCC1 was preferentially localized to the periphery of p40-positive spheres, whereas other invasion- and drug resistance-related markers, including ABCC2, ABCG2 and MMP-2, were not detected. These findings indicate that the peripheral expression of MMP-14 and ABCC1 does not reflect a generalized stress response, but rather a selective phenotypic feature associated with squamous metaplasia and spatial heterogeneity in 3D PDAC spheres.

Pathway analysis implicated numerous signaling pathways in epithelial plasticity and squamous differentiation, which may contribute to this process. Notably, pathway analysis of proteomic data identified enrichment of transcriptional regulation-related and histone acetylation-associated pathways, suggesting the involvement of epigenomic regulation in squamous differentiation in PDAC. These findings provide a rationale for future studies to focus on epigenetic regulators, such as chromatin-modifying enzymes, as potential drivers of lineage plasticity and squamous transdifferentiation. Pathways associated with the ‘integrin family’, extracellular matrix remodeling and the ‘intermediate filament signaling pathway’ were commonly enriched, supporting the notion that cell-cell and cell-matrix interactions serve a central role in initiating squamous differentiation under 3D culture conditions. In addition, transcriptional regulation by p63, a marked regulator of squamous epithelial lineage commitment, was consistently enriched in PDAC cell lines capable of expressing squamous markers. This supports the notion that squamous differentiation in PDAC cells is regulated at the transcriptional level rather than exhibiting a non-specific phenotypic alteration. The spatial confinement of p40 expression to the sphere periphery further suggests that local microenvironmental cues, such as mechanical stress, cell density or paracrine signaling, may modulate p63-driven transcriptional programs (44). Therefore, no subsequent direct inhibition experiments were conducted, however a number of the present results provide mechanistic support for proposing such experiments. This finding was further supported by the spatially limited expression of p40 at the sphere periphery under 3D culture conditions, suggesting localized activation of p63-driven transcriptional programs. These results indicate that squamous differentiation in PDAC cells is regulated at the transcriptional level rather than representing a non-specific phenotypic change and provide a biologically plausible rationale for future inhibition studies targeting p63-mediated transcriptional regulation. Future studies should therefore aim to investigate the inhibition of candidate proteins involved in integrin signaling, cytoskeletal remodeling or p63-mediated transcription to potentially regulate squamous differentiation in PDAC.

Numerous signaling pathways previously implicated in squamous differentiation, including NOTCH, TGF-β and EGFR signaling, are modulated by cell-cell interactions and 3D tissue organization (45–48). Although direct inhibition or functional pathway analyses were not performed in the present study, the enrichment of pathways associated with intercellular interaction, transcriptional regulation and cytoskeletal remodeling is consistent with the involvement of these regulatory axes in squamous differentiation under 3D culture conditions. In addition, enrichment of histone acetylation-related pathways suggests that epigenetic regulation may contribute to the observed phenotypic plasticity, as chromatin remodeling is implicated in lineage switching and squamous transdifferentiation in numerous carcinomas (49,50).

Notably, the differential expression and spatial localization of squamous markers under 2D and 3D culture conditions could have direct clinical implications for patients with PDAC. Specifically, the acquisition or regional enrichment of squamous markers, such as CK5/6 and p40, in 3D cultures, may reflect the phenotypic plasticity associated with tumor aggressiveness, therapeutic resistance and poor prognosis, which are characteristic features of ASC (51). These findings suggest that conventional 2D cultures may underestimate squamous differentiation, whereas 3D culture systems may exhibit improved mimicking of the *in vivo* tumor microenvironment relevant to diagnosis and drug response. The expression of squamous markers by specific PDAC cell lines in 3D culture demonstrates the utility of this *in vitro* model for investigating the biological behavior of ASC and testing therapeutic strategies targeting squamous-like PDAC phenotypes. The present study suggests that a number of the squamous components observed in PDAC tissues and ASCs were formed by the differentiation of PDAC cells into cancer cells with squamous components. The regulatory mechanisms of squamous cell component expression in PDAC cell lines, particularly with regard to epigenetic regulation, require further investigation. Immunocytochemical staining of p63 alone showed positive results in both 2D- and 3D-cultured MIA PaCa-2 cells, however p40 staining was negative and no increase was demonstrated by proteomics. Therefore, it is unlikely that squamous epithelial components are expressed in MIA PaCa-2 cells.

The present study has several limitations. Although eight PDAC cell lines, including five epithelial-type and three mesenchymal-type lines, were analyzed, this panel may not fully represent the heterogeneity of squamous metaplasia observed in PDAC. In addition, the analyses were largely descriptive and no formal statistical comparisons were performed, warranting cautious interpretation of the observed differences. Furthermore, immunocytochemical staining was evaluated qualitatively without quantitative assessment of staining intensity or positive cell ratios, which limits direct comparison across cell lines and culture conditions. Future studies incorporating a larger panel of cell lines, statistical analyses, and quantitative image-based evaluation will be required to strengthen the robustness and generalizability of the findings.

In conclusion, the present study demonstrated that 3D culture conditions markedly affected both the expression levels and spatial distribution of squamous cell markers in PDAC cell lines. This suggests that squamous differentiation in PDAC is regulated by extracellular signals from outside the cells. Yet, additional studies are required to further elucidate the regulation of the expression of squamous cell components in PDAC cells.

## Supplementary Material

Supporting Data

Supporting Data

## Data Availability

The data generated in the present study may be found in the jPOST repository under accession number JPST004363 or at the following URL: https://repository.jpostdb.org/entry/JPST004363.
