# Supplementary material for: Proteomic and immunocytochemical analyses of squamous components in two- and three-dimensional-cultured pancreatic ductal adenocarcinoma cell lines
Source: Oncol Lett. 2026 Mar 19;31(5):185. doi: 10.3892/ol.2026.15540 (PMC13034107; doi:10.3892/ol.2026.15540)

Figure S1. Immunocytochemical analysis of CK20 in PDAC cell lines. The localization of CK20 in PDAC cell lines cultured 2D and 3D cultures was examined by immunocytochemical staining with a specific antibody. Neither the 2D nor 3D cultures showed CK20-positive cells. The DLD-1 human colorectal cancer cell line cultured in 2D and 3D conditions severed as positive control. Scale bars, 50 μm. CK, cytokeratin; 2D, two-dimensional; 3D, three-dimensional; PDAC, pancreatic ductal adenocarcinoma.

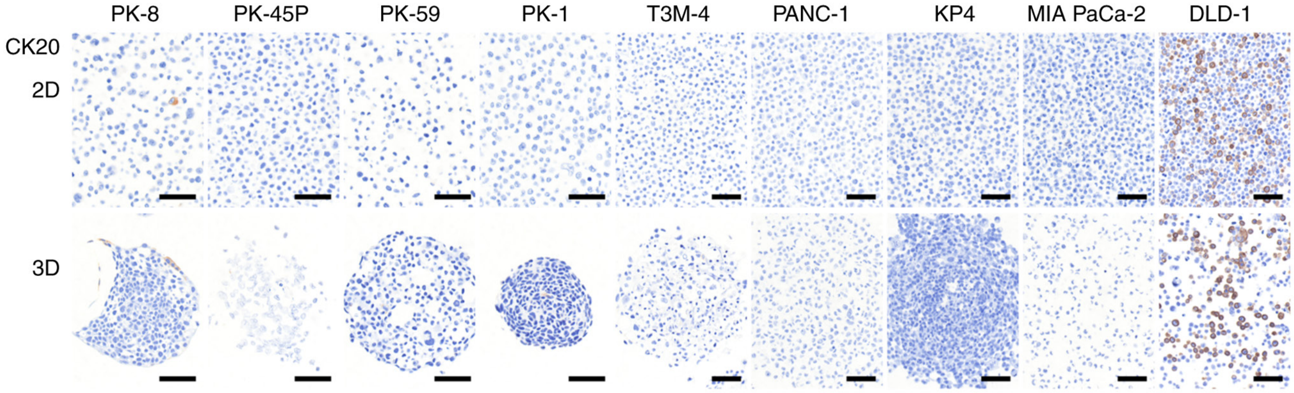

Figure S2. Proteomic analysis of CK6B and CK6C in PDAC cell lines. The expression of CK6B and CK6C in eight PDAC cell lines cultured in 2D and 3D cultures was examined using proteomic analysis. The expression levels of CK6C were below the detection limit in PK-45P, PANC-1, KP4 and MIA PaCa-2 cells under 2D culture conditions and in PANC-1, KP4 and MIA PaCa-2 cells under 3D culture conditions. CK, cytokeratin; 2D, two-dimensional; 3D, three-dimensional; PDAC, pancreatic ductal adenocarcinoma.

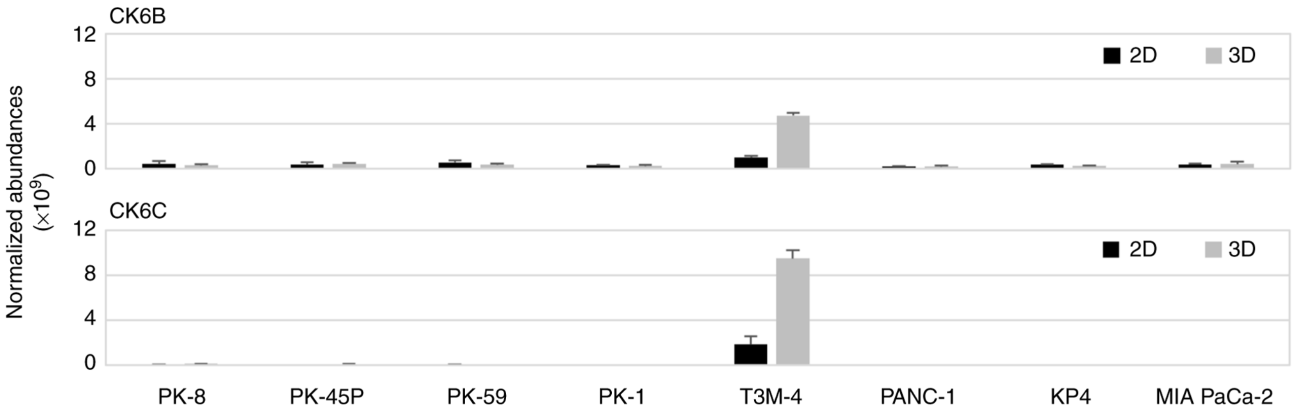

Figure S3. Immunocytochemical analysis of ABCC1 and MMP-14 in PDAC cell lines. The localization of ABCC1 and MMP-14 in PDAC cell lines cultured in three-dimensions was examined through immunocytochemistry using a specific antibody. ABCC1 immunoreactivity was observed throughout the entire spheres in both cell types, with particularly strong staining observed in peripheral cells. By contrast, MMP-14 showed weak immunoreactivity predominantly in cells located at the sphere periphery. By contrast, ABCC2, ABCG2 and MMP-2 showed no detectable immunoreactivity in either the sphere core or peripheral regions. Scale bar, 50 μm. ABCC, ATP binding cassette subfamily C; PDAC, pancreatic ductal adenocarcinoma; MMP, matrix metalloproteinase; ABCG, ATP-binding cassette sub-family G.

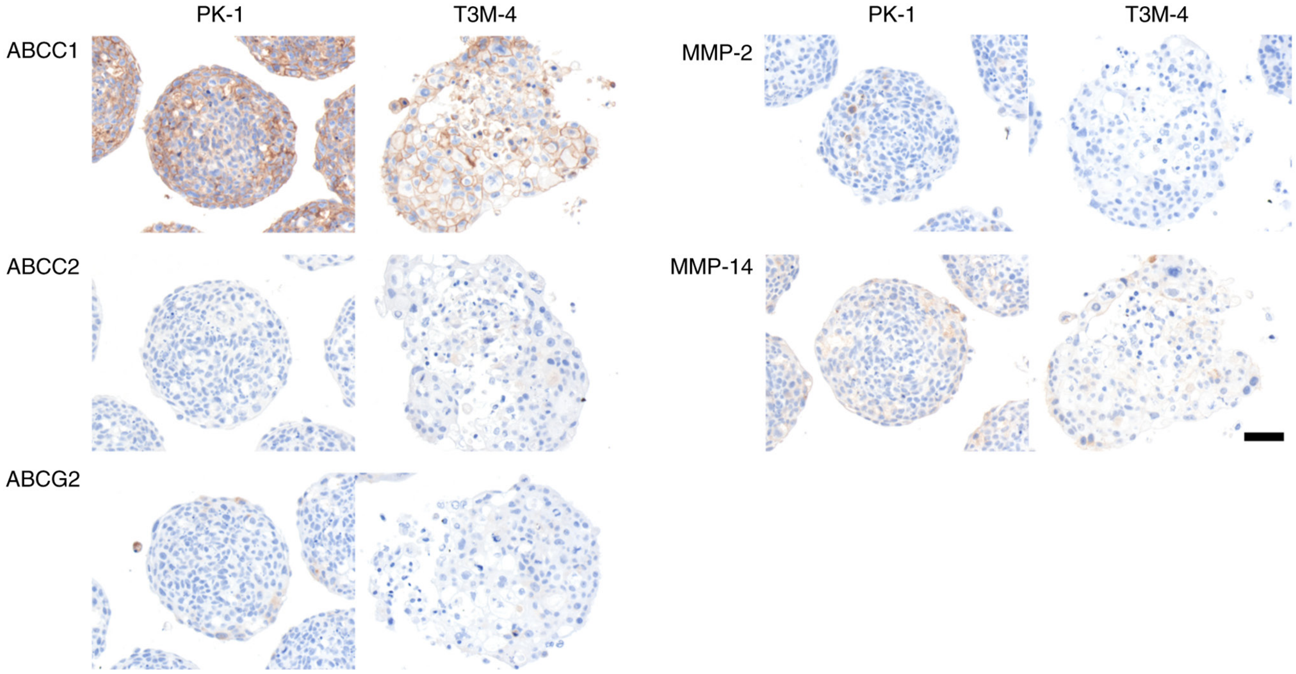

Supplement: Supporting Data [file Supplementary_Data1.pdf]
